# Spontaneous Eyeblinks Are Correlated with Responses during the Stroop Task

**DOI:** 10.1371/journal.pone.0034871

**Published:** 2012-04-06

**Authors:** Jihoon Oh, Mookyung Han, Bradley S. Peterson, Jaeseung Jeong

**Affiliations:** 1 Department of Bio and Brain Engineering, Korea Advanced Institute of Science and Technology (KAIST), Daejeon, Republic of Korea; 2 College of Medicine, The Catholic University of Korea, Seoul, Republic of Korea; 3 Department of Psychiatry, College of Physicians and Surgeons, Columbia University, New York, New York, United States of America; Tokyo Metropolitan Institute of Medical Science, Japan

## Abstract

The timing and frequency of spontaneous eyeblinking is thought to be influenced by ongoing internal cognitive or neurophysiological processes, but how precisely these processes influence the dynamics of eyeblinking is still unclear. This study aimed to better understand the functional role of eyeblinking during cognitive processes by investigating the temporal pattern of eyeblinks during the performance of attentional tasks. The timing of spontaneous eyeblinks was recorded from 28 healthy subjects during the performance of both visual and auditory versions of the Stroop task, and the temporal distributions of eyeblinks were estimated in relation to the timing of stimulus presentation and vocal response during the tasks. We found that the spontaneous eyeblink rate increased during Stroop task performance compared with the resting rate. Importantly, the subjects (17/28 during the visual Stroop, 20/28 during the auditory Stroop) were more likely to blink before a vocal response in both tasks (150–250 msec) and the remaining subjects were more likely to blink soon after the vocal response (200–300 msec), regardless of the stimulus type (congruent or incongruent) or task difficulty. These findings show that spontaneous eyeblinks are closely associated with responses during the performance of the Stroop task on a short time scale and suggest that spontaneous eyeblinks likely signal a shift in the internal cognitive or attentional state of the subjects.

## Introduction

A large number of human studies have shown that the spontaneous eyeblink rate (EBR) varies with changes in the cognitive state [1]. In adults, for example, the mean EBR increases significantly during the spontaneous generation of speech and while listening to and memorizing passages of speech, compared with the EBR during quiet rest [2]. EBRs also increase during the silent recall of both verbal and pictorial stimuli, whereas they decrease when attending to passive verbal or pictorial stimuli [3]. During spontaneous conversation, which requires no memorization, the EBR doubles, on average, compared with that during quiet rest. In contrast, reading aloud reduces the EBR by nearly half over quiet rest or gaze fixation [4], [5]. Moreover, the EBR declines during the performance of tasks that require sustained visual attention, such as the visual tracking of moving stimuli [3], [6], the recollection of numbers in a working memory task, or while daydreaming [7].

Studies of temporal distributions of eyeblinks under visual stimulus conditions have revealed that spontaneous eyeblinks are distributed non-uniformly during the task with a close correlation to the stimulus [8], [9], [10], [11]. For example, during the reading of written text, a large proportion of eyeblinks occur at or near the end of a line of the text, before the gaze returns to the beginning of the next line [12]. When subjects performed the discrimination task of presented stimuli, the eyeblinks were distributed mainly after the stimulus presentation in both the visual and auditory conditions [9]. These findings suggest that the eyeblinks may signal the end of one cognitive process, the beginning of another or the shift of one of these processes to the other.

These results suggest that certain cognitive processes might be responsible for the alteration of the EBR. Attentional requirements and concentration on visual stimuli have been thought to be the crucial factors affecting the EBR [13], [14]. However, alterations in EBR might not be solely related to the processing of visual information because various internal events that are not relevant to visual stimuli also modulate the EBR. Cognitive load and operational memory, which require no visual information, increased the EBR [3], [7]. The activity of other cognitive processes, such as the binding of visual and action features [15] and the size of attentional blink [16], were also related to the rate of eyeblinking.

In addition to these cognitive processes, physiological state is also known to modulate the occurrence of eyeblinking. Sympathetic arousal and relaxation affect the long-term rates of eyeblinking [17], and visual fatigue caused by prolonged work modified some features of spontaneous eyeblinking [18]. Because eyeblinks are mainly induced by the contraction of facial muscles (the orbicularis oculi muscle), vocalization that utilizes other facial muscles (the orbicularis oris muscle) is also thought to affect the occurrence of eyeblinking [2], [19]. Previous studies that used verbal responses have reported a significant increase in EBR during tasks [2], [3], but these studies have not indicated the relationship between the timing and temporal pattern and verbalization. Thus, it is unclear whether the eyeblinks are facilitated by the verbalization itself or by the aroused state during the task [3].

Despite the emphasis of previous studies on defining the changes in EBR under various cognitive or behavioral states, the timing or temporal patterns of eyeblinks under certain cognitive processes are still poorly understood. Thus, we aimed to study how the frequency and timing of eyeblinks vary with specific cognitive processes during an attentional task. We investigated the temporal patterns of eyeblinks during the performance of visual and auditory Stroop tasks; in particular, we estimated the timing of eyeblinks with respect to the timing of stimulus presentation and vocal response. If the eyeblinks were associated with a particular cognitive process or a change in one of the processes during the task, then the pattern of eyeblinks might be related to the timing of certain cognitive processes. In this study, we found that the temporal distributions of eyeblinks during the task was non-uniform, thus providing evidence that eyeblinks are linked temporally, and potentially causally, to specific cognitive processes during this attentional task.

The Stroop [20] task is one of the most widely used paradigms to study attention and cognitive control. In this task, the participants name the color of the ink (red, yellow, blue, green) in which the words are written. The words themselves either name colors or objects that are unrelated to the colors, and the words grouped into three stimulus conditions: (1) congruent, in which the word names the color that matches the ink in which the word is written; (2) conflict, in which the word names a color other than the ink in which the word is written; and (3) neutral, in which the word names an object that is unrelated to the color. The identification of ink colors for congruent stimuli is faster and therefore produces shorter reaction times (RTs) compared with the identification of colors for neutral stimuli. This phenomenon is called Stroop “facilitation.” The naming of ink colors for conflict stimuli is delayed and thus produces longer RTs compared with the naming of colors for neutral or congruent stimuli. This effect is called Stroop “interference.” This interference is attributed by many to the greater automaticity of word reading than color naming, requiring greater cognitive control and a greater allocation of attentional resources, and therefore, results in delayed responses to avoid erring on the task [21], [22].

We selected the Stroop task to study eyeblinks because each trial of the task includes an implicit series of cognitive processes, including perception, attentional allocation, decision-making, and motor response, which we hope will facilitate inferences made concerning the functional role of eyeblinks during specific cognitive processes during the task. The subjects performed both visual and auditory versions of the Stroop task to determine whether the stimulus modality was uniquely associated with the observed eyeblink dynamics.

## Materials and Methods

### Ethics Statement

All of the subjects were provided with written informed consent for the study, which was approved by the Institutional Review Board of the Korea Advanced Institute of Science and Technology (KAIST).

### Subjects

Twenty-eight healthy, right-handed subjects (13 men, 15 women) 20–27 years of age (23.5 ± 1.7, mean ± s.d.) were recruited from the undergraduate population of four universities in the town of Daejeon, South Korea. The exclusion criteria were the following: (1) evidence of identifiable cognitive impairment as assessed by questionnaires, (2) the use of contact lenses or eye glasses, and (3) the use of any medications based on self-report. None of the subjects had previously participated in electroencephalogram (EEG) or electrooculogram (EOG) recording experiments. Before the experiment, to avoid influencing the timing or frequency of eyeblinks through knowledge that we were recording the timing of their eyeblinks, the subjects were informed that the aim of the study was to obtain EEG recordings. When questioned at the end of the experiment, none of the subjects acknowledged awareness or suspicion that this experiment was recording eyeblinks.

### The Stroop Tasks

**Figure 1:**
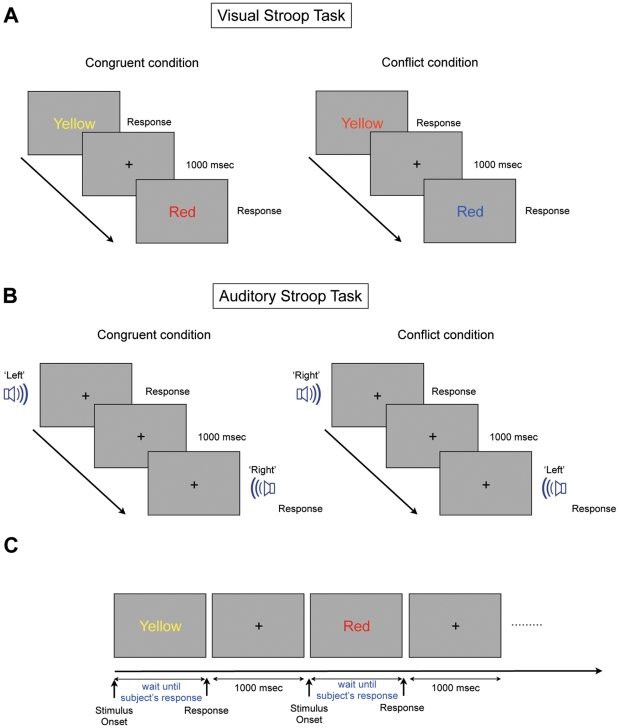
The Visual and Auditory Stroop Paradigms. For the visual paradigm, the subjects were asked to name the color of the word in the color naming task and to read the name of the word in word reading task. Following the subject’s response and prior to the presentation of the next stimulus, a white fixation cross-hair was presented for 1000 msec. (A) Visual Stroop task. In the visual Stroop task, the subjects received a word that was either congruent, conflicting or neutral, and these types were presented in a constant ratio. The congruent stimulus is presented in the same color name of its own color, and the conflict stimulus is presented in a different color name of its own color. The neutral stimulus consists of four different words that are not related to the meaning of the color in which it is written. (B) Auditory Stroop task. The structure of the auditory Stroop task was similar to the visual Stroop task except for the stimulus condition. The word ‘left’ emanating from the left side speaker consisted of a congruent stimulus, while the word ‘left’ emanating from the right side speaker consisted of a conflict stimulus. Unlike the visual Stroop task, the fixation point was presented during both the stimulus and resting conditions to induce the subject’s eye fixation. (C) The structure of one set in the visual Stroop task. Each stimulus was presented until the subject’s response. The reaction time of all subjects was ranged from about 500 msec to 1,500 msec.

The single word presentation version of the original Stroop task [20] was used (Figure 1). We presented two different sets (congruent dominant set, conflict dominant set), and two conditions, a word reading condition (word repetition in auditory Stroop) and a color naming condition (direction naming in auditory Stroop), were applied to each set. During the word reading condition, the subjects were asked to make a rapid vocal response to the name of the word presented, and during the color naming condition, they were asked to respond rapidly with the color in which the word was written and not with the name of the object that the word denoted. Similarly, under the word repetition condition in the auditory Stroop, subjects were required to make a rapid vocal response to the repetitious word emanating from the each speaker. In contrast, under the direction naming condition in the auditory Stroop, the subjects were told to respond rapidly with the direction in which the word was emanating and not with the name of the object that the word denoted. The order of the stimuli was randomly allocated within the ratio allocation described above.

In the visual version of the Stroop task, four color-denoting words (“red”, “yellow”, “blue”, and “green”) and four neutral words (“chair”, “sorrow”, “pencil”, and “telephone”) were presented to the subject in the same proportion of each color (red, yellow, blue, or green, respectively) (Fig. 1). The congruent stimuli consists of words denoting colors that matched the color in which the words were presented (e.g., “red” written in red). The conflict stimuli consists of words denoting colors other than the color of the ink in which the words were presented (e.g., “blue” written in red). The neutral stimuli consists of words denoting objects other than color, but were presented in one of four colors (e.g., “table” written in red). In addition, the subjects performed four different versions of the visual Stroop (word reading-congruent, word reading-conflict, color naming-congruent, and color naming-conflict).

In the auditory version of the Stroop task [23], [24], the stimuli consists of four words denoting spatial directions (“front”, “rear”, “left”, and “right”). These were presented from either speaker that was positioned either to the left, right, front, or rear of the subject. The congruent stimuli consists of words denoting a direction that matched the direction from which the word was presented (e.g., the word “left” was presented from the left speaker). The conflict stimuli consists of words denoting a direction other than the direction from which the word was presented (e.g., the word “right” presented from the left speaker). The neutral stimuli consists of words denoting objects other than direction, but were presented in one of four directions (e.g., “table” presented from the left speaker). The subjects were instructed to gaze at a fixation point located at the monitor during both the visual and auditory Stroop tasks and performed four different versions of the auditory Stroop task (word repetition-congruent, word repetition-conflict, direction naming-congruent, direction naming-conflict) because eyeblinks tend to occur when subjects shift their gaze [25].

The subjects were instructed to maintain their gaze fixation on the cross-hair for 2 minutes following every 2 stimuli sets, which was used to determine the baseline EBR. We termed this data as the resting condition and it was used as a control for the EBR.

Each of the stimuli was presented with different durations (575 ± 170 msec, mean ± s.d., ranged 350∼1490 msec), and terminated upon the subject’s verbal response. Therefore, the stimulus durations varied over trials because of individual variability in the reaction times. The subject’s response was represented by a button press because it produces larger interference effects in behavioral studies [22]. When the subjects failed to respond or uttered an inaudible response, the next stimulus was automatically presented 2 seconds following the onset of the last stimulus. Inter-stimulus intervals were 1,000 msec. Each stimulus set (conflict or congruent) consisted of 60 trials, with a mean duration of 2 minutes. Both of the visual and auditory versions of the task consisted of two different sets of stimuli (congruent and conflict). The congruent-dominant set consists of 60 trials: 42 (70%) congruent, 12 (20%) conflict, and 6 (10%) neutral trials. The conflict-dominant set consists of trials of the same ratio as the congruent set, but with 42 (70%) conflict, 12 (20%) congruent, and 6 (10%) neutral trials. The order of stimuli in one set was randomly allocated within the ratio allocation as described above. In total, four visual sets (in the following order: word reading condition of set A, word reading condition of set B, color naming condition of set A, and color naming condition of set B) and four auditory stimulus sets (in the following order: word repetition condition of set A, word repetition condition of set B, direction naming condition of set A, and direction naming condition of set B) were presented to each subject, equaling an experimental duration of 60 minutes, which included experimental setup.

### Testing Environment

The testing was performed in a sound-proof room where the EEG equipment was located and where the subjects were positioned in the middle of the four speakers (front, rear, left, and right). The distance from the speakers to the subject was 1.0 m. A 19-inch LCD monitor was located 0.7 m in front of the subject and displayed word stimuli that subtended 15 degrees of the subject’s field of view. The height of the chair in which the subject sat was adjusted so that the monitor and speakers were at the same height as the subject’s eyes and ears. Because excessively loud sounds were likely to cause a reflex eyeblink, the noise level of the speakers was adjusted to the comfort level of each subject before the experiment began. The room humidity was held constant at approximately 50% to ensure that drying of the cornea did not influence the rate of the eyeblinks.

### Data Acquisition

We used the vertical electrooculography (vEOG) channel of Neuroscan’s 64-channel EEG acquisition system (Neuroscan Inc. VA, USA) to record the occurrence timing of the eyeblinks. In the vEOG data, we were able to discriminate eyeblink and eye movement. The eyeblinks were represented by a narrow peak in the vEOG data [26]; however, the eye movements were observed to alter the baseline of the data and exhibited similar patterns with a stepwise function. Thus, we could discriminate between eyeblinks and ocular rotation from the vEOG data. Because the subjects were required to fixate their eyes on the cross-hair, only a few (<1% of the total number of eyeblinks) eye movements were found during the visual and auditory Stroop tasks.

We also recorded the EEG during the performance of the Stroop task to discourage awareness that this was an assessment of eyeblinks. The EEG cap was placed on the subject’s head and the vEOG electrode was place 10–15 mm above the upper eyelid and 20 mm below the lower margin of the eyelid. All of the experimental procedures were recorded with a video camera (Sony HDC-1, Japan, 30 frames/sec).

We classified non-spontaneous eyeblinks from the vEOG data in two different ways. First, we automatically measured the onset-time of potential in spontaneous blinks using the program (MATLAB 7.9, *MathWorks, USA)*. In this process, eyeblinks that exhibited amplitudes of more than 4 standard deviations of the average eyeblink amplitude for each subject were automatically discarded. Then, we manually investigated all of the raw vEOG data to find non- spontaneous blinks based on distinct eye closures. In the vEOG raw data, we could clearly discern non-spontaneous blinks because they demonstrated larger amplitudes and durations than spontaneous blinks [26]. If there were non- spontaneous blinks in each session, we eliminated them from the table of onset-time, which was acquired by the program.

The vocal responses were recorded in two ways. The data from the microphone was stored in a PC, and wave files was extracted from the videotape recorders’ video file (HDC-1, Sony, Japan). We used the STIM2 hardware (Neuroscan Inc. VA, USA) to synchronize the vEOG and the vocal file. The STIM2 hardware produced time cues whenever the buttons were pressed and these cues were recorded in the vEOG data. Simultaneously, silent beep sounds produced by the stimuli-presenting program were recorded in the wave file. At the initiation of each trial and each stimulus set, a silent beep and the cue signal for the vEOG were presented simultaneously by STIM2. These were then used as synchronization markers for the vEOG and vocal recording.

### Cross-correlogram and Statistical Analyses

A cross-correlogram visually represents the synchrony between the events in a time series (e.g., between eyeblinks and the timing of stimuli or responses in this study). It is most often plotted as a histogram in which the height, position, and number of peaks represent the temporal relationship between eyeblinks and behavioral events of interest (e.g., stimulus presentation, vocal response). The peaks before, during, or after the time point t  =  0 indicate that eyeblinks tend to occur before, at, or after the behavioral event of interest, respectively.

The distribution of the vEOG data during the trials of the Stroop task was estimated using a cross-correlogram. The temporal differences between the eyeblink and vocal response were measured using the vEOG data and record of response timing for each subject. Of all of the 11,210 trials acquired during the visual and auditory Stroop tasks, 3,768 trials (33% of the total trials) had two eyeblinks in a trial. The mean interval between these two eyeblinks was 656 ± 71 msec (mean ± s.d.) in the visual Stroop and 889 ± 90 msec in the auditory Stroop tasks. Since these blinks occurred in close proximity with each other, the second eyeblink predominately occurred during the last half of the delay period (82% in the visual Stroop and 91% in the auditory Stroop). We calculated in the cross-correlogram that all of the eyeblinks occurred in each trial including successive eyeblinks. The bin size used to calculate the cross-correlogram was 30 msec.

We determined the subgroups through the parameters of eyeblink distributions, which was the peak location of the histogram. From the histogram of each session, we measured the location of peak before or after the vocal response. If all of the sessions had peaks before the vocal response, we classified the subject as belonging to subgroup I. Similarly, if all of the sessions had peaks after the vocal response, the subject was classified as belonging to subgroup II. Subjects were placed in subgroup III if they had a peak before the vocal response in certain sessions and after the vocal response in others.

The data were analyzed using the SPSS 11.0 software. The presence of Stroop effects when comparing RTs for word reading (word repetition in the auditory Stroop) with color naming (direction naming in the auditory Stroop) and when comparing RTs for conflict with congruent conditions were assessed using a two-way ANOVA and Student’s t-test. The comparisons of EBR for word reading (word repetition in the auditory Stroop), color naming (direction naming in the auditory Stroop), or for conflict and congruent conditions was evaluated using a one-way ANOVA. Student’s T-test assessed the differences in the eyeblink peak locations during word reading (word repetition in the auditory Stroop) and color naming (direction naming in the auditory Stroop) and during the conflict and congruent sets. The statistical thresholds were conservatively set with two-tailed probability values of *p* < 0.01 to help minimize Type I errors associated with multiple statistical tests.

## Results

### Visual Stroop Task

#### Behavioral data

A repeated measures ANOVA (including two factors for word reading vs. color naming, and two factors for conflict and congruent stimuli) demonstrated a significant difference in the RTs between word reading and color naming (word reading 484 ± 41 msec; color naming 696 ± 111 msec, F (1,108)  =  236.74, *p* < 0.001). There was also a main effect of congruency (conflict 616 ± 155 msec; congruent 565 ± 106 msec; F(1,108)  =  13.3, *p* < 0.001) and this effect showed that RTs in congruent stimuli were faster than those in conflict stimuli. Finally, we found an interaction between the congruency and stimulus conditions (F(1, 108)  =  14.6, *p* < 0.001). These results indicate the presence of the Stroop interference effect.

#### Task-related changes in EBR

**Figure 2:**
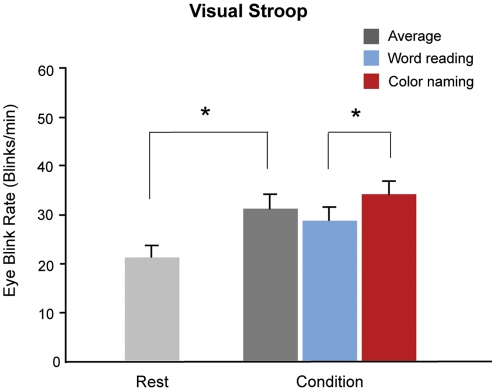
Eyeblink rates (EBR) during the task. The EBR had increased significantly during the task condition. The histogram shows the mean EBR in the resting state and Stroop task condition. In the resting condition, the subjects were required to fixate their eye on to the fixation point for 2 minutes, and the EBR was 20.7 ± 11.2 blinks/min. The EBR in the task condition was measured during the Stroop task and was 30.7 ± 13.0 blinks/min. (error bars denote standard error).

The EBR in task-demand states was significantly higher than that in resting states (F(1,54)  =  13.8, *p* < .001, one-way ANOVA) (Fig. 2). In the resting state, the average EBR was 20.7 ± 11.2 blinks/min, increasing to 30.7 ± 13.0 blinks/min during the Stroop task. The EBR increased in the word reading compared with the color naming condition (33.4 ± 12.6 blinks/min vs. 27.9 ± 12.9 blinks/min, T_104_  =  2.21, *p*  =  0.02), indicating that the frequency of the eyeblinks varied with cognitive load.

#### Cross-correlogram for vocal responses and eyeblinks

The mean RT during the visual Stroop was 588 ± 172 msec (mean ± s.d.). Although 22.9% (1,257/5,475 trials) of all of the trials were unaccompanied by any eyeblink, and 12.4% (680/5,475) of the trials were accompanied by more than two eyeblinks, 64.7% (3,538/5,475) of the trials were associated with only a single blink. The mean number of eyeblinks per trial in the visual Stroop task was 0.8 ± 0.4.

Upon visual inspection, we found that most eyeblinks were present in temporal proximity to the verbal response in both versions of the task. Statistical analyses also showed that eyeblinks were more closely distributed to vocal response compared with stimulus onset. The eyeblinks were closer to the vocal response than the stimulus onset (172 ± 103 msec vs. 645 ± 187 msec) and the standard deviation of the blink distribution was larger in the stimulus onset than that of the vocal response (vocal response: 367 ± 108 msec, stimulus onset: 686 ± 195 msec; one-way ANOVA, F(1,54)  =  44.8, *p* < 0.001). This result suggests that eyeblinks are more correlated with vocal response than with stimulus onset in the visual Stroop task.

**Figure 3:**
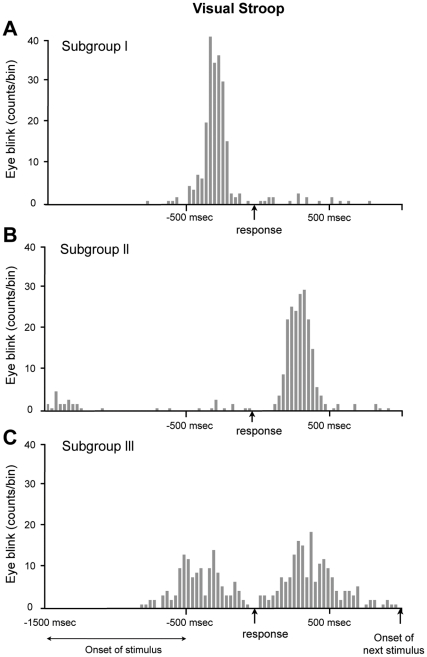
A representative example of a subject from each subgroup. We classified 28 subjects into 3 subgroups based on the position of the eyeblink peak location. The histogram (having 30 msec intervals) was drawn based on the time difference between the eyeblink and visual response for 240 trials per subject. (A) Subgroup I. The subjects who blinked mostly before the vocal response were classified as belonging to subgroup I. Of the 28 subjects, 17 belonged to this group. The mean number of eyeblinks was 1.1 ± 0.3 blinks/trial and the eyeblinks demonstrated a distribution of 210 ± 85 msec. (B) Subgroup II. The subjects who had a peak location after the vocal response were classified as belonging to subgroup II. Of the 28 subjects, 7 belonged to this group. The mean number of eyeblinks per a trial was 0.9 ± 0.4 blinks/trial and the peak location was 380 msec with standard deviation of 116 msec. (C) Subgroup III. The subjects who blinked both before and after the vocal response were classified as belonging to subgroup III. Of the 28 subjects, 4 belonged to this group. This bimodal distribution was drawn because the subject blinked before the response in one set, but blinked after the response in another set. Relative to subgroups I and II, subgroup III had a lower peak value due to its two different peak locations.

For all of the trials except for those in which no eyeblink had occurred, the time interval between the eyeblink and vocal response yielded a cross-correlogram demonstrating a peak prior to the response in 17 subjects, indicating that most of the eyeblinks occurred immediately preceding the vocal response (Fig. 3A). The mean number of eyeblinks per trial was 1.1 ± 0.3 (approximately 90% of the trials were accompanied by a single eyeblink).

A considerable number of subjects (7/28, labeled “Subgroup II”) blinked following the verbal response. A typical example is shown in Fig. 3B. This subject showed an average time delay of 380 ± 116 msec. The mean number of eyeblinks per trial was 0.9 ± 0.4 (85% of the trials were accompanied by a single eyeblink). A third type of subject (“Subgroup III”, detected in 4/28 subjects) had a bimodal distribution of eyeblinks around the time of the response (Fig. 3C), indicating that the eyeblinks occurred either before or after the response, or both. This subject, on average, blinked 0.7 ± 0.6 times per trial (65% of the trials were accompanied by a single eyeblink). Thus, most of the eyeblinks were distributed in the vicinity of the Stroop response, rather than near the onset of the stimulus. Moreover, the subjects could be classified into three groups based on the timing of their eyeblinks with respect to the timing of their responses.

#### Peak locations for eyeblink distributions

**Figure 4:**
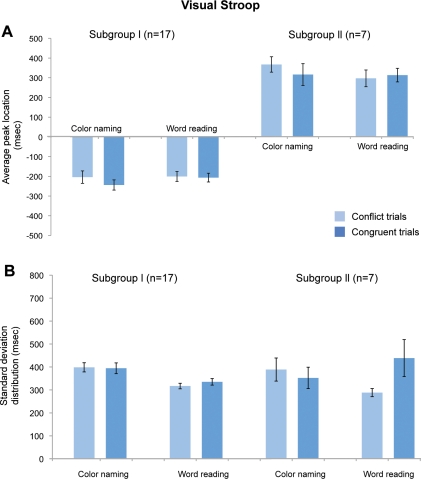
Average peak location and standard deviation distribution for all subjects. No significant differences were found between the stimulus type and condition. (A) For the 28 subjects, their peak locations were measured in each set, and the mean and standard deviation value across all of the subjects are presented. The total average peak location is the mean value of the subject’s entire set of peak values and is measured from the 30 msec interval histogram, which consists of 120 trials. The conflict and congruent average peak location is the subset of the total average peak location that is derived from each stimuli, which consists of 60 trials. The minus sign means that the peak was located before the response. As seen in the histogram, most subjects demonstrate a peak value near 250 msec before the response in subgroup I and 300 msec after the response in subgroup II. (B) The standard deviation distribution is the mean value of the standard deviation from the peak value in each subject with error bars showing the standard errors across all of the subjects.

The location of the peak in the eyeblink histograms relative to the Stroop response in Subgroup I (17/28 subjects) was -232 ± 109 (mean ± s.d.) msec for color naming and -225 ± 100 msec for word reading, and this difference was not significant (T(32, 2.04)  =  0.19, *p*  =  0.84) (Fig. 4). The peak location did not differ for the stimulus type in each stimulus condition (conflict vs. congruent in color naming, -204 ± 122 msec vs. -244±101 msec, T(32, 2.04)  =  1.06, *p*  =  0.29; conflict vs. congruent in word reading -200 ± 89 msec vs. -206 ± 104 msec, T(32, 2.04)  =  0.69, *p*  =  0.49). Our findings were similar for Subgroup II (7/28 subjects): the average peak location was 338 ± 177 msec for color naming and 310 ± 174 msec for word reading (T(12, 2.18)  =  1.05, *p*  =  0.31, two- tailed Student’s unpaired t-tests). For stimulus type in each stimulus condition, the findings were as follows: conflict vs. congruent in color naming, 367 ± 139 msec vs. 316 ± 236 msec, T(12, 2.18)  =  0.87, *p*  =  0.40; conflict vs. congruent in word reading 297 ± 173 msec vs. 313 ± 121 msec, T(12, 2.18)  =  0.11, *p*  =  0.92; both two-tailed Student’s unpaired t-tests. Subgroup III was excluded from this analysis because their bimodal distribution did not have a common peak value. These findings indicated that the patterns of eyeblinking in Subgroups I and II, with respect to the timing of the Stroop response, were consistent across stimulus type (i.e., conflict or congruent stimuli) and task difficulty (i.e., word reading or color naming conditions).

We also investigated the task performances between Subgroup I and Subgroup II. Reaction times were not significantly differ in these two groups (subgroup I vs. subgroup II in color naming, 708±114 msec vs. 685 ± 105 msec, T(8,2.30)  =  1.25, *p*  =  0.25; in word reading, 484 ± 39 msec vs. 473 ± 35 msec, T(8,2.30)  =  0.72, *p*  =  0.48; Student’s t-tests). The correction rate, which measures the ratio of correct answers to total number of trials, was not significantly different between the two groups (subgroup I vs. subgroup II, 96% vs. 94%, respectively; T(8,2.30)  =  0.13, *p*  =  0.89, Student’s t-tests).

### Auditory Stroop Task

#### Behavioral data

A repeated measures ANOVA for RTs in the auditory version of the task that included two factors for word repetition vs. direction naming and two factors for condition (conflict vs. congruent) demonstrated a significant difference in the RTs between word repetition and direction naming (word repetition 531 ± 81 msec; direction naming 700 ± 116 msec, F(1,108)  =  52.51, *p* < 0.001) and between the stimulus conditions (conflict, 641 ± 143 msec; congruent, 589 ± 112 msec; F(1,108)  =  3.24, *p*  =  0.06). These findings again indicate the presence of the Stroop interference effect.

#### Task-related changes in EBR

**Figure 5:**
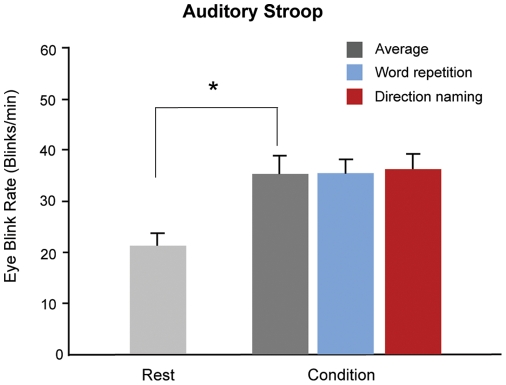
Eyeblink rates (EBR) during the task. The histogram shows the mean EBR in the resting state and Stroop task condition (with error bars showing the standard errors). In addition, the EBR had increased significantly during the Stroop task condition. In the resting condition, the subjects were required to fixate their eye on to a fixation point for 2 minutes, and the EBR was 20.7 ± 11.2 blinks/min. The EBR in the task condition was measured during the Stroop task and was 35.0 ± 14.2 blinks/min. This EBR increase in the task condition relative to the resting state is consistent with the result of visual Stroop tasks. (Error bars denote standard errors).

For a total of 112 stimulus sets in 28 subjects, we measured the mean eyeblink rate to confirm that the EBR is affected by stimulus condition. As in the visual Stroop task, the EBR in task-demand states (word repetition, direction naming conditions) was significantly higher than that in resting states. (F(1,54)  =  22.8, *p* < 0.0001; one-way ANOVA) (Fig. 5). In the resting state, the average EBR was 20.7 ± 11.2 (mean ± s.d.) blinks/min, increasing to 35.2 ± 14.2 blinks/min during the auditory Stroop task. Unlike the visual task, the EBR did not differ significantly between the stimulus conditions (word repetition, 35.1 ± 14.2 blinks/min; direction naming, 35.3 ± 15.8 blinks/min). This change in the EBR during the auditory Stroop was similar to the change detected during the visual Stroop, and no significant difference was found between them (33.4 ± 12.6 blink/min in visual Stroop, 35.2 ± 14.2 blinks/min in auditory Stroop, F(1,54)  =  0.61, *p*  =  0.43, one-way ANOVA)

#### Cross-correlograms for vocal response and eyeblinks

We estimated the histogram for eyeblinks in all of the subjects. All trials (a total 5735 trials) were classified by the number of eyeblinks per trial. We found that 12.3% (705/5735) of all trials were unaccompanied by any eyeblink, 36.4% (2088/5735) of the trials were accompanied by more than two eyeblinks, and 51.3% (2942/5735) of the trials were associated with only one eyeblink. The mean number of eyeblinks per trial in the auditory Stroop was 1.3 ± 0.5 (the EBR was 1.0 ± 0.5 blinks/trial in both tasks). The cross-correlogram was drawn to determine the relationship between the eyeblink and response timing for all of the trials except for 12.3% of the total block, which had no eyeblink.

Most of the eyeblinks occurred in temporal proximity to the verbal response on the auditory task. Similar to the visual Stroop task, the eyeblinks were distributed closer (138 ± 93 msec vs. 741_± 170 msec) and narrower to the vocal response (vocal response: 504 ± 158 msec vs. stimulus onset: 859 ± 232 msec; one-way ANOVA, F(1,54)  =  56.76, *p* < 0.001).

**Figure 6:**
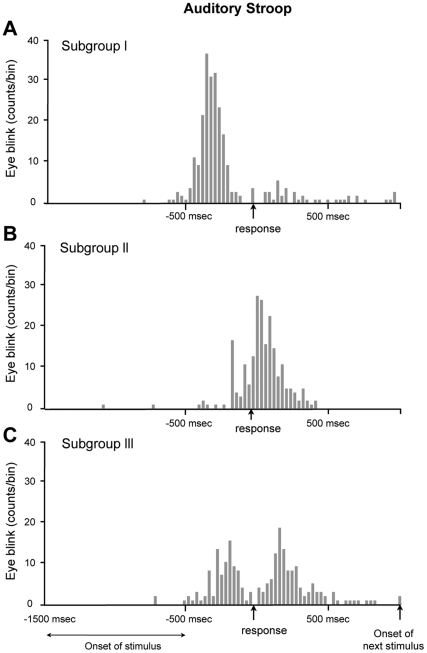
A representative example of a subject from each subgroup. We classified the 28 subjects into 3 subgroups based on the position of eyeblink peak location. The histogram (having 30 msec intervals) was drawn based on the time difference between the eyeblink and visual response for 240 trials per subject. (A) Subgroup I. The subjects who blinked mostly before the vocal response were classified as belonging to subgroup I, and 20 of 28 subjects belonged to this subgroup. The mean number of eyeblinks was 1.2 ± 0.6 blinks/trial and had a distribution of 240 ± 95 msec. (B) Subgroup II. The subjects who had a peak location after the vocal response were classified as belonging to subgroup II, and 2 of 28 subjects belonged to this group. The mean number of eyeblinks per trial was 0.9 ± 0.5 blinks/trial and the peak location was 340 msec with a standard deviation of 98 msec. (C) Subgroup III. The subjects who blinked before and after the vocal response were classified as belonging to subgroup III, and 6 of 28 subjects belonged to this group. This bimodal distribution was drawn because the subject blinked before the response in one set but blinked after the response in another set. Relative to subgroup I and II, this subgroup had a lower peak value due to its two different peak locations.

For all of the trials except for those in which no blink had occurred, the time interval between the eyeblink and vocal response yielded a cross-correlogram that showed a peak prior to the response in 20 of the 28 subjects, indicating that most of the eyeblinks occurred immediately preceding the vocal response (Fig. 6A). These subjects blinked an average of 1.2 ± 0.6 blinks/min (75% of trials were accompanied by a single blink) and exhibited a peak value at 240 ± 95 msec before the response.

Only a minority of the subjects (2 of 28, labeled “Subgroup II”) blinked following the verbal response during the auditory task, A representative example is displayed in Fig. 6B. This subject had an average time delay of 340 ± 98 msec and at an average rate of 0.9 ± 0.5 blinks per trial (79% of the trials were accompanied by a single blink). A third type of subject (“Subgroup III”, representing 6 of the 28 subjects) had a bimodal distribution of eyeblinks, which was centered around the time of the vocal response (Fig. 6C), indicating that eyeblinks occurred either immediately before or after the response, or both. This subject, on average, blinked 0.8 ± 0.7 times per trial (61% of the trials were accompanied by a single blink). Thus, most of the eyeblinks were distributed in the vicinity of the Stroop response and not near the onset of the stimulus. Moreover, similar to the visual task, the subjects could be classified into three groups by the timing of their blinking with respect to the response: only before, only after, or either before or after the response (Fig. 6).

#### Peak locations for eyeblink distributions

**Figure 7:**
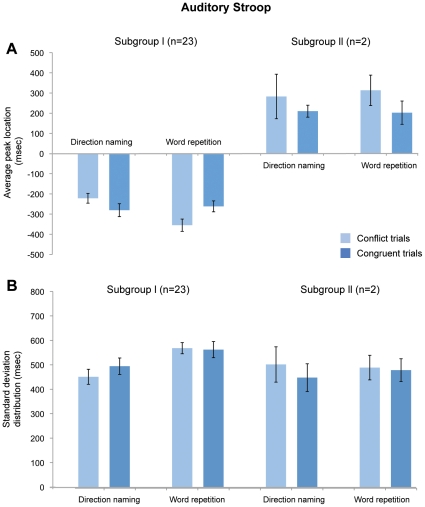
Average peak location and standard deviation distribution for all subjects. No significant differences were found between the stimulus type and condition as in the visual Stroop task. (A) For the 28 subjects, peak locations were measured in each set, and the mean and standard deviation value across all of the subjects are presented. The total average peak location is the mean value of the subject’s entire set of peak values and is measured from the 30 msec interval histogram, which consists of 120 trials. The conflict and congruent average peak location is a subset of the total average peak location that is derived from each stimuli, which consists of 60 trials. (B) The standard deviation distribution is the mean value of the standard deviation from the peak value in each subject with error bars showing standard errors across all of the subjects.

The location of the peak in the eyeblink histograms relative to the verbal response in Subgroup I (20/28 subjects) was -270 ± 130 msec for direction naming and -275 ± 123 msec for word repetition (Fig. 7). However, the timings did not differ significantly from one another (T(38, 2.02)  =  0.04, *p*  =  0.97). The peak location also did not differ for stimulus types in each stimulus condition (conflict vs. congruent in direction naming -282 ± 329 msec vs. -210 ± 130 msec, T(38, 2.02)  =  1.44, *p*  =  0.16; conflict vs. congruent in word repetition -313 ± 174 msec vs. -202 ± 177 msec, T(38, 2.02)  =  1.24, *p*  =  0.22). Subgroups II and III, which contain 2 and 6 subjects, respectively, were excluded from these analyses because of their small sample size.

In both of the visual and auditory versions of the task, the eyeblinks were related closely in the timing of the vocal response, rather than with the onset of the stimulus. Subgroup I consisted of subjects who blinked primarily before the response, in particular, during the auditory Stroop tasks (20 of 28 subjects).

## Discussion

This study is the first quantitative investigation of the timing of eyeblinks during performance of the visual and auditory Stroop task. The EBR increased in all of the subjects in temporal proximity to the Stroop response, regardless of the stimulus modality (visual or auditory) and task difficulty (congruent or conflicting stimuli). In the majority of the subjects, the eyeblinks occurred 150–250 msec before the vocal response in both of the visual and auditory tasks. In a minority of the subjects, the eyeblinks occurred, on average, 200 msec following the vocal response, and in an even smaller subgroup, the eyeblinks occurred either immediately before or after the response.

Because our Stroop design involves multiple cognitive processes in a short trial (i.e., the onset of stimulus, internal processing of the Stroop effect, offset of Stroop stimuli and vocal response in a trial), we focused on two discrete internal events, the stimulus onset and the vocal response, in analyzing the relationship between the timing of eyeblinks and cognitive processes. These two processes could be distinguished from others because their exact timing was recorded in each trial. The stimulus onset was separated by an inter-stimulus interval of 1,000 msec from the previous trial. Thus, the possible presence of the Stroop effect or other cognitive processes in previous trials might be diminished at the time of the stimulus onset. In both visual and auditory Stroop tasks, eyeblinks were more closely and significantly associated with the vocal response than the stimulus onset. These findings strongly suggest that spontaneous eyeblinks are closely associated with responsive behaviors during this task, likely during a change in the cognitive set, such as during decision making or the shift from sustained attention to the stimuli to a motor response within a short time. These findings strongly suggest that spontaneous eyeblinks are closely associated with responsive behaviors during this task, likely during a change in the cognitive set, such as during decision-making or the shift from sustained attention to the stimuli to a motor response within a short time scale.

The EBR increased during performance of the Stroop task compared with the EBR during the resting condition, which is consistent with prior reports that EBR increases during the performance of tasks that require sustained attention [3], [27]. The EBR, however, can also decrease during the performance of certain attentional tasks, such as silent reading [28], or during certain tasks of sustained visual attention [13]. These disparate findings may potentially be understood as reflecting a greater EBR during tasks of sustained attention that are composed of multiple discrete trials, such as the Stroop tasks employed in the present study, rather than a reduced EBR during attentional tasks that consists of essentially only a single trial, or at least a relatively small number of trials, such as prolonged silent reading. If eyeblinks signal some sort of response readiness, or perhaps a readiness for the next trial (e.g., when eyeblinks occur following the verbal response during the Stroop task), then the EBR would naturally be expected to increase during multi-trial tasks relative to a resting baseline. Tasks that are relatively unitary and sustained, such as silent reading, do not involve frequent changes between stimulus processing and response readiness or shifts in the cognitive set from one trial to another, and, therefore, these tasks would be expected to decrease the EBR relative to the resting baseline when attentional shifts during free association are frequent.

Previous studies have shown that the EBR increased during certain cognitive states, such as memorizing sentences [2] and speaking [28], but decreased during reading [4]. This alteration in the EBR during the cognitive processes is thought be related to the cognitive load of the tasks. In a study using visual stimuli, the EBR decreased as the stimulus difficulty increased [28]. Moreover, the EBR increased when the subjects were required to silently rehearse the presented visual stimuli [3]. These results suggest that visual attention might influence the EBR in certain cognitive processes.

The similar findings obtained in the auditory and visual Stroop tasks in this study suggest that the role of the eyeblink during an attentional task is one that generalizes across stimulus modalities. Thus, an increased EBR during the Stroop task and peak eyeblink distribution near the vocal response might be related to the processing of Stroop-related performance rather than the stimulus modality. In performing the Stroop task, a series of cognitive processes are needed; [22] the processing of perceived stimuli, the interference between cognitive processes and then vocal responses must occur.

For both versions of the Stroop task used in this study, each trial consisted of at least three stages of information processing: (i) from the onset of the stimulus to the decision to make a vocal response (500–1500 msec), which is a stage requiring a considerable allocation of sustained attention; (ii) from the time when the decision to respond is made until completion of the response (80–150 msec); and (iii) from the completion of the response to the beginning of the next trial (1 sec). In this study, most of the eyeblinks occurred during the first two stages of information processing, despite the fact that the average trial duration in both of the tasks was approximately two seconds, much shorter than the average EBR at rest. These observations strongly suggest that eyeblinking is in some way involved in, or at least is a marker of, a cognitive process that the tasks engage.

However, the result of absence of congruency in both the visual and auditory Stroop tasks also suggests that peak eyeblink distribution near the vocal response might be related to the processing of the response rather than the processing of the perceived stimuli or interference effects. These kinds of correlations between the vocal response and eye blinking could arise from the similar motor activities for the eyelids and speech [2]. A previous study found a relationship between the EBR and speech motor activity and suggested that topographically adjacent motor channels could cause concurrent activation in the eyeblink and speech [2]. fMRI studies reported that the orbitofrontal cortex was activated during spontaneous eye blinking and the primary motor cortex and medial frontal cortex were activated during voluntary eye blinking [29], [30]. Although the major muscles used in eye blinking and motor speeches have different cranial nerve origin [19], these results suggest that the instant initiation of vocal speech could affect the occurrence of eyeblinks.
